# Comparing the 7th and 8th editions of UICC/AJCC staging system for nasopharyngeal carcinoma in the IMRT era

**DOI:** 10.1186/s12885-021-08036-8

**Published:** 2021-03-30

**Authors:** Tao He, Ruo-Nan Yan, Hua-Ying Chen, Yuan-Yuan Zeng, Zhong-Zheng Xiang, Fang Liu, Bian-Fei Shao, Jia-Chun Ma, Xi-Ran Wang, Lei Liu

**Affiliations:** 1 Department of Head and Neck Oncology, Cancer Center, State Key Laboratory of Biotherapy, and the Department of Radiation Oncology, West China Hospital, Sichuan University, No. 37 Guo Xue Alley, Chengdu, Sichuan PR China; 2 Department of Radiation Oncology, Cancer Center, The Sixth Floor Of The Third Inpatient Building Of West China Hospital Of Sichuan, West China Hospital, Sichuan University, Chengdu, Sichuan PR China

**Keywords:** Nasopharyngeal carcinoma, Simultaneous integrated boost, Intensity-modulated accelerated radiotherapy, UICC/AJCC staging system, Prognosis, Radiotherapy

## Abstract

**Background:**

To compare the prognostic value of 7th and 8th editions of the Union for International Cancer Control/American Joint Committee on Cancer (UICC/AJCC) staging system for patients with nonmetastatic nasopharyngeal carcinoma (NPC) treated with intensity-modulated radiotherapy and simultaneous integrated boost– intensity-modulated radiation therapy (SIB-IMRT).

**Methods:**

Patients with NPC (*n* = 300) who received SIB-IMRT were included. Survival by T-classification, N-classification, and stage group of each staging system was assessed.

**Results:**

For T-classification, nonsignificant difference was observed between T1 and T3 and between T2 and T3 disease (*P* = 0.066 and 0.106, respectively) for overall survival (OS) in the 7th staging system, whereas all these differences were significant in the 8th staging system (all *P <* 0.05). The survival curves for disease-free survival (DFS) and locoregional recurrence-free survival (LRRFS) in both staging systems were similar, except for the comparison of T2 and T4 disease for LRRFS (*P* = 0.070 for 7th edition; *P* = 0.011 for 8th edition). For N-classification, significant differences were observed between N2 and N3 diseases after revision (*P* = 0.046 and *P* = 0.043 for OS and DFS, respectively). For staging system, no significant difference was observed between IVA and IVB of 7th edition.

**Conclusion:**

The 8th AJCC staging system appeared to have superior prognosis value in the SIB-IMRT era compared with the 7th edition.

**Supplementary Information:**

The online version contains supplementary material available at 10.1186/s12885-021-08036-8.

## Introduction

Nasopharyngeal carcinoma (NPC) develops in the epithelial lining of the nasopharynx, the narrow tubular passage behind the nasal cavity, and radiation therapy (RT) is the primary treatment because of anatomical constraints and high radiosensitivity of this carcinoma.

Over the past 2 decades, NPC management has undergone substantial changes. Magnetic resonance imaging (MRI), which has been widely used in the clinical staging of NPC, has made it possible to define tumor volume precisely and allow the early detection of occult metastases [1–3]. In terms of disease modality, intensity-modulated radiation therapy (IMRT) could decrease the overall treatment time and increase the fractionation dose to planned target volume (PTV) with relatively less late toxicity compared with 2-dimensional conventional or 3-dimensional conformal radiotherapy [4–8]. Therefore, the new staging system should be based on up-to-date clinical data and maintain its relevance with current management approaches.

The classification of N3 in the 7th edition of the Union for International Cancer Control /American Joint Committee on Cancer (UICC/AJCC) NPC staging system is mainly based on anatomy, and its ability to predict prognosis is limited [9, 10]. The UICC/AJCC released the 8th edition of this staging system in an attempt to further help clinicians opt for the best treatment for patients. The revised staging system was mainly derived from a study on 1609 patients with NPC on the basis of their MRI findings and IMRT status [11]. A few key revisions in the 8th edition are as follows: (1) for T-classification, patients with infratemporal fossa/masticator space involvement, which was considered in the T4 category in the 7th edition, has been replaced with a precise definition; (2) medial pterygoid (MP) and lateral pterygoid (LP) muscles have been downgraded to the T2 category, whereas prevertebral muscles have been included in the T2 category; (3) For the N-classification, the supraclavicular fossa (SCF) has been replaced by the caudal border of the cricoid cartilage, and N3a and N3b in the 7th edition have been reclassified to N3; (4) T4 and N3 have been merged into IVA; (5) For the clinical stage, stages IVA and IVB in the 7th edition have been redefined as IVA, and stage IVC has been reclassified as IVB in the 8th edition. The supplement shows the classification criteria of the 7th and 8th editions of the UICC/AJCC nasopharyngeal carcinoma staging system.

Patients with NPC can receive IMRT via 2 push modes: the conventional sequential boost and simultaneous integrated boost. Compared with the conventional method, SIB-IMRT can be delivered in different target regions during the same treatment session and has a shorter waiting time. In addition, most clinical data revealed that SIB-IMRT had better sparing of the parotid glands and inner ear structures [12, 13].

We performed this retrospective study to explore the clinical outcomes of SIB-IMRT, and compare the 7th and 8th editions of the UICC/AJCC staging system.

## Materials and methods

### Patient characteristics

A total of 300 patients with newly diagnosed, pathologically proven, non-distant metastatic NPC who were treated with SIB-IMRT at West China Hospital between February 2009 and December 2013 were included in our study. Table 1 summarizes the characteristics of all the patients. All the patients had no tumor history and did not receive any radiotherapy previously. The number of men was 215 (71.7%), whereas the number of women was 85 (28.3%). Median age was 47 years (range, 11–81 years). All patients received the following pretreatment evaluations: recording of completed patient history, haematological and biochemical profiles, physical examination, flexible fiberoptic endoscopic examination, MRI of the nasopharynx and neck, abdominal sonography, chest radiography or CT, and whole-body bone scan. All the patients were reclassified according to the 7th and 8th editions of the UICC/AJCC staging system by 2 clinicians. A third clinician was consulted to reach a consensus in case of disagreement.

**Table 1:** Patient characteristics

Characteristic	N(%)
Sex	
Male	215 (71.7%)
Female	85 (28.3%)
Age (year)	
< 45	126 (42%)
45–60	131 (43.7%)
≥ 60	43 (14.3%)
CT	
IC	173 (57.7%)
CC	67 (22.3%)
CA	42 (14%)
NO CT	18 (6%)
Histopathology	
WHO I	1 (0.3%)
WHO II	299 (99.7%)

*Abbreviations*: *CT* Chemotherapy. *IC* Induction+ concurrent chemotherapy. CC Concurrent chemotherapy. *CA* Concurrent + adjuvant chemotherapy

### RT

All patients completed radical SIB-IMRT at the Tumor Center of West China hospital according to the guidelines for RT based on reduced volume IMRT [14]. RT is carried out in accordance with the guidelines of NCCN radiotherapy for NPC. The primary nasopharynx gross tumor volume (GTVnx) and metastatic cervical lymph nodes (GTVnd) included all gross diseases observed in the MRI scan (radiotherapy physician mainly based on the fusion of contrast-enhanced MRI and planning CT after induction chemotherapy, also referred to contrast-enhanced MRI and planning CT before induction). CTV-1 was defined as a high-risk region that included the primary nasopharynx tumor volume with a 5–10 mm margin and the entire nasopharynx. CTV-2 was defined as potentially involved region that included the skull base, pterygopalatine fossa, pterygoid processes, anterior third of the clivus and cervical vertebra, inferior sphenoid sinus and cavernous sinus, nasopharyngeal cavity (including the posterior region of the nasal cavity), maxillary sinus (5 mm anterior to the maxillary mucosa and posterior nares), posterior ethmoid sinus, parapharyngeal space, and bilateral retropharyngeal lymph nodal regions. The clinical target volume of the neck node regions (CTV-N) covered Levels II, III, IV, and V, prophylactic coverage of ipsilateral Level Ib lymph node level in CTVn2 (intermediate prophylactic dose) if there is: disease involvement of the submandibular gland or involvement of structures that drain to level Ib as the first echelon site (namely the oral cavity, anterior half of nasal cavity) or involvement of level II LNs with extracapsular extension. According to the recommendation of the Radiation Therapy Oncology Group (RTOG)/European Organization for Research and Treatment of Cancer delineation consensus. SIB-IMRT was administered for 1 month after the patients completed induction chemotherapy. The radiation doses delivered were 70 Gy to GTVnx and GTVnd in 33 fractions at 2.12 Gy per fraction, 60 Gy at 1.81Gy per fraction to CTV-1, and 56 Gy to CTV-2 and CTV-N in 33 fractions, 5 times per week.

### Chemotherapy

A total of 173 patients received both induction chemotherapy and concurrent chemotherapy (cisplatin 80 mg/m^2^ divided into 3 parts on days 1–3, every 3 weeks). The main induction chemotherapy regimen was TPF (paclitaxel 135 mg/m^2^ day 1, cisplatin 80 mg/m^2^ divided into 3 parts on days 1–3, and fluorouracil 750 mg/m^2^ per day on days 1–5, every 3 weeks). Of the total, 67 patients received only concurrent chemotherapy and 42 patients received concurrent chemotherapy along with the adjuvant chemotherapy, and the main adjuvant chemotherapy regimen was TPF and PF (cisplatin 80 mg/m^2^ divided into 3 parts on days 1–3, and fluorouracil 750 mg/m^2^ per day on days 1–5, every 3 weeks).

### Follow-up

The patients were followed up every 3 months during the first 3 years and every 6 months thereafter or until death. Each follow-up consisted of physical examination, basic serum chemistry, flexible fiberoptic endoscopy, MRI of the nasopharynx and neck, chest radiography or CT, abdominal sonography, and a whole-body bone scan. The above examinations were performed after SIB-IMRT to detect locoregional or distant relapse.

### Statistical analysis

The endpoints of this study were overall survival (OS; time to death due to any cause), disease-free survival (DFS; time to treatment failure or death from any cause), distant failure-free survival (DMFS; time to distant metastasis), and locoregional recurrence-free survival (LRRFS; time to locoregional persistence or recurrence). All the events were estimated from historical diagnosis. OS, DFS, DMFS, and LRRFS were calculated using the Kaplan–Meier method [15], and survival curves were estimated using log-rank tests [15]. Statistical Package for the Social Sciences, version 23.0, was used for statistical analysis.

## Results

### T category classification

Of the 89 patients with stage T4 NPC according to the 7th edition, 61 were downgraded to T3 considering the 8th edition as the cancer had reached to the medial or lateral pterygoid. A total of 2 patients with stage T1 NPC according to the 7th edition were reclassified as stage T2 considering the 8th edition on the basis of prevertebral muscle extension (Table 2).

**Table 2:** The patient distribution of both two stages system

7th edition						8th edition					
T category	Number	N category	Number	Clinical stage	Number	T category	Number	N category	Number	Clinical stage	Number
T1	82	N0	50	I	18	T1	80	N0	50	I	18
T2	62	N1	82	II	50	T2	64	N1	82	II	50
T3	67	N2	141	III	126	T3	128	N2	123	III	166
T4	89	N3a	8	IVA	79	T4	28	N3	45	IVA	66
		N3b	19	IVB	27						

**Fig. 1:**
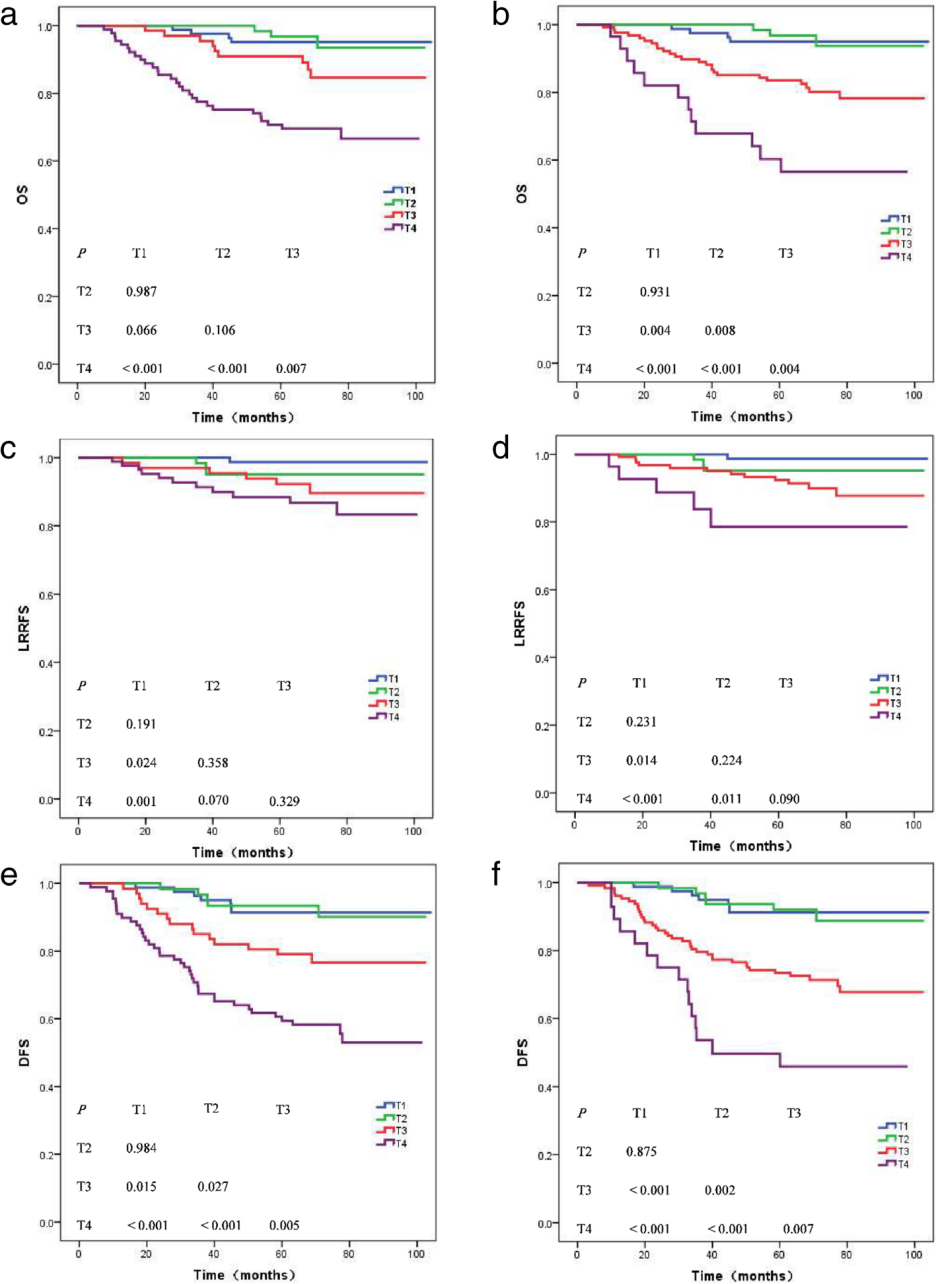
Overall survival (**a**, **b**), locoregional recurrence-free survival (**c**, **d**), and disease-free survival (**e**, **f**) for each T categories in the 7th (**a**, **c**, **e**) and 8th editions (**b**, **d**, **f**)

Table 3 presents the 5-year survival rates for different end points of T categories in the 7th and 8th editions. Figure 1a and b show the OS curves for the T categories in the 7th and 8th editions. There were significant differences between T4 and T1, T4 and T2, and T4 and T3 categories in the 7th and 8th editions (*P <* 0.001 for T4 and T1, T4 and T2; *P* = 0.007 for T4 and T3 in the 7th edition; *P* = 0.004 for T4 and T3 in the 8th edition). The OS rates between T3 and T2, and T3 and T1 categories in the 7th edition were not significantly different (*P* = 0.106 and *P* = 0.066, respectively), whereas these categories in the 8th edition were considerably different (*P* = 0.008 and *P* = 0.004, respectively). Figure 1c and d show the LRRFS rates of T categories in both the staging systems. In the 8th edition, the difference between T2 and T4 was statistically significant, while that in the 7th edition was not (*P* = 0.070 and *P* = 0.011, respectively). Therefore, the 8th edition had improved the prognosis value of NPC compared with the.

**Table 3:** Survival rates at 5-year

7th edition				8th edition			
T category	OS	DFS	LRRFS	T category	OS	DFS	LRRFS
T1	95.1%	91.5%	98.8%	T1	95.0%	91.3%	98.7%
T2	93.5%	90.1%	95.1%	T2	93.7%	88.8%	95.2%
T3	84.6%	76.6%	89.6%	T3	81.4%	72.6%	91.4%
T4	69.5%	58.2%	83.3%	T4	56.5%	50.0%	78.5%
N category	OS	DFS	DMFS	N category	OS	DFS	DMFS
N0	97.0%	97.0%	98.0%	N0	97.0%	97.0%	98.0%
N1	84.4%	75.3%	92.3%	N1	87.8%	75.3%	92.3%
N2	84.4%	75.8%	88.4%	N2	84.8%	77.1%	89.0%
N3a	75.0%	75.0%	75.0%	N3	72.3%	61.5%	78.5%
N3b	71.1%	50.5%	74.8%				
Clinical stage	OS	DFS		Clinical stage	OS	DFS	
I	100.0%	100.0%		I	100.0%	100.0%	
II	98.0%	98.0%		II	98.0%	98.0%	
III	91.3%	88.7%		III	88.0%	78.9%	
IVA	74.6%	70.2%		IVA	73.4%	61.5%	
IVB	74.6%	72.2%					

*Abbreviations*: *OS* Overall survival. *DFS* Disease-free survival. LRRFS Locoregional recurrence-free survival. *DMFS* Distant metastasis-free survival

7th edition. Figure 1e and f show the DFS curves; except for the difference between T1 and T2 categories, the difference between all other combinations of classifications were statistically significant.

### N category classification

In the 8th edition, the supraclavicular fossa (SCF) was replaced with the lower neck (below the caudal border of the cricoid cartilage), which led to the upstaging of 18 patients from N2 to N3 (Table 2).

**Fig. 2:**
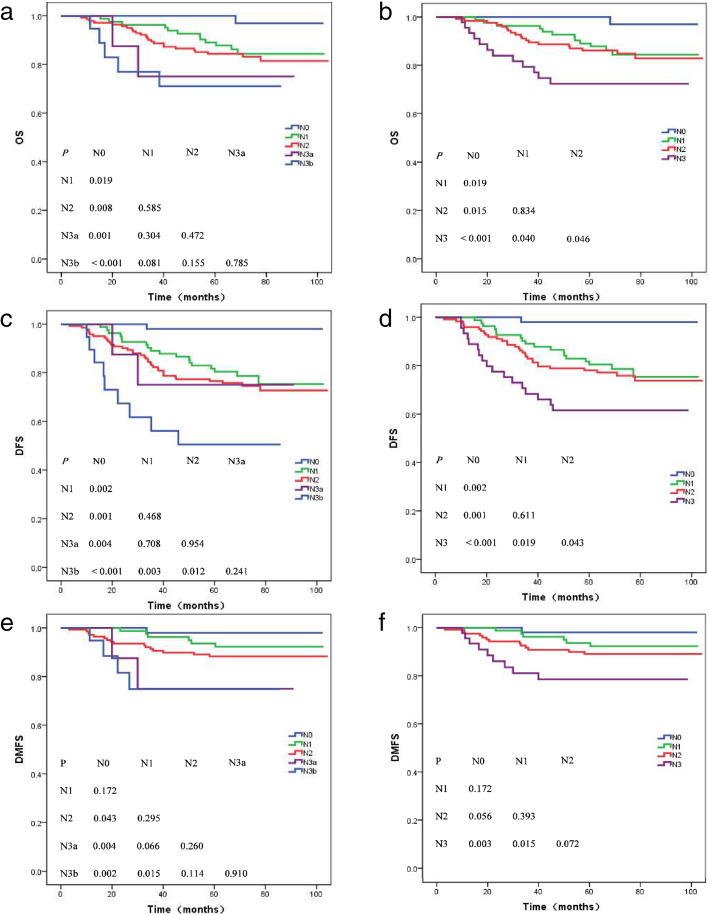
Overall survival (**a**, **b**), disease-free survival (**c**, **d**), and distant failure-free survival (**e**, **f**) for each N categories in the 7th (**a**, **c**, **e**) and 8th editions (**b**, **d**, **f**)

Table 3 presents the 5-year survival rates for different end points of N categories in the 7th and 8th editions. Figure 2 shows the OS, DFS, and DMFS survival curves for each staging system. The OS and DFS in the 7th edition system for N2 and N3a stages were not significantly different (*P* = 0.472 and *P* = 0.954, respectively, Fig. 2a, c), whereas the OS and DFS for N2 and N3 using the 8th edition were statistically different (*P* = 0.046 and *P* = 0.043, respectively, Fig. 2b, d). Thus, the 8th edition had a superior prognosis value compared with the 7th edition with respect to N category classification. Additionally, there was no significant difference between classifications N3a and N3b in the 7th edition system (*P* = 0.785 for OS, *P* = 0.241 for DFS, and *P* = 0.910 for DMFS; Fig. 2a, c, e). The DFS curves for N3a and N3b even overlapped in the 7th edition (Fig. 2c). Therefore, merging N3a and N3b stages in the 8th edition was reasonable.

### Stage group classification

**Fig. 3:**
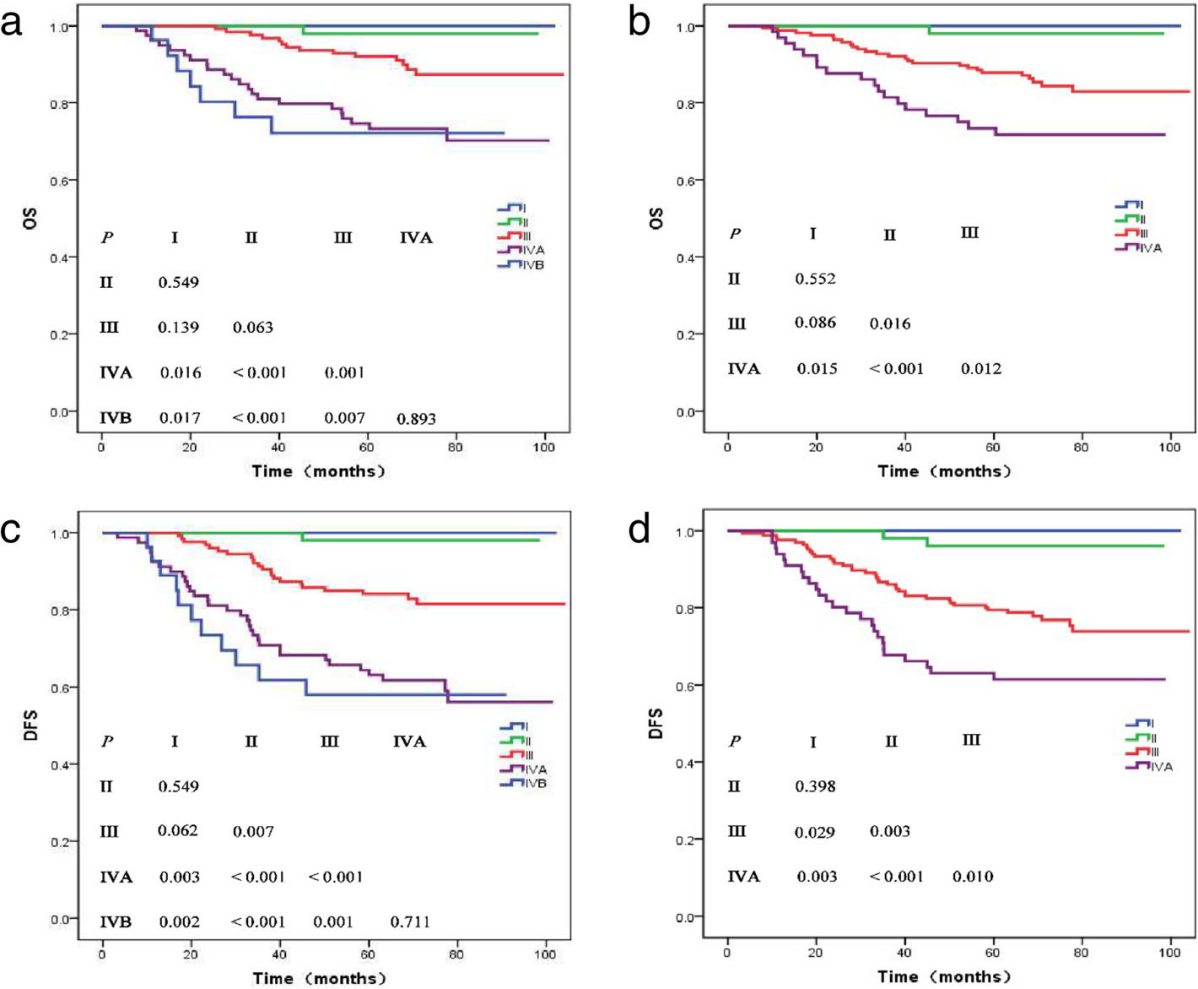
Overall survival (**a**, **b**) and disease-free survival (**c**, **d**) for each clinical stage in the 7th (**a**, **c**) and 8th editions (**b**, **d**)

Considering the 8th edition, 40 patients with stage IV disease were downgraded to stage III (Table 2). No deaths were reported for stage I patients. Table 3 presents the 5-year survival rates for different end points of clinical stages in the 7th and 8th editions. Figure 3 shows the OS and DFS survival curves for each staging system. In both the staging systems, significant differences in OS and DFS were observed for clinical stages (*P <* 0.05) except for stages IVA and IVB, and I and II (*P* = 0.893 for OS and *P* = 0.711 for DFS; *P* = 0.549).

### Multivariate analyses

To further discover prognostic factors, three factors were involved in multivariate analysis: age (<45 vs. ≥45), sex (male vs. female) and T category or N category. Table 4 shows the results of multivariate analysis by adjusted age, sex and T category or N category of the two editions. The results indication differences of hazard of deaths between T1 and T2, N1 and N2 were non statistical significance.

**Table 4:** Multivariate analysis by adjusted age, sex and T category or N category of the two editions

	OS		LRRFS		DFS	
7th edition						
T category	HR (95%CI)	*P-*value	HR (95%CI)	*P-*value	HR (95%CI)	*P-*value
T1 vs. T2	1.465 (0.350–6.133)	0.601	1.114 (0.352–3.525)	0.855	1.183 (0.450–3.111)	0.733
T1 vs. T3	0.559 (0.178–1.715)	0.318	1.513 (0.383–5.975)	0.554	0.614 (0.269–1.399)	0.245
T1 vs. T4	0.212 (0.081–0.558)	0.020	1.583 (0.456–5.502)	0.470	0.305 (0.149–0.625)	0.001
Overall *P*	<0.001		0.364		<0.001	
8th edition						
T category	HR (95%CI)	*P-*value	HR (95%CI)	*P-*value	HR (95%CI)	*P-*value
T1 vs. T2	1.506 (0.360–6.307)	0.575	1.190 (0.376–3.768)	0.767	1.075 (0.424–2.726)	0.878
T1 vs. T3	0.343 (0.130–0.908)	0.031	1.470 (0.510–4.238)	0.476	0.487 (0.239–0.996)	0.049
T1 vs. T4	0.194 (0.065–0.580)	0.003	0.310 (0.030–3.203)	0.326	0.239 (0.100–0.572)	0.001
Overall *P*	<0.001		0.144		<0.001	
	OS		DMFS		DFS	
7th edition						
N category	HR (95%CI)	*P-*value	HR (95%CI)	*P-*value	HR (95%CI)	*P-*value
N1 vs. N0	0.104 (0.013–0.805)	0.300	0.781 (0.138–4.410)	0.779	0.137 (0.032–0.595)	0.008
N1 vs. N2	0.862 (0.427–1.743)	0.680	0.580 (0.184–1.823)	0.351	0.944 (0.533–1.671)	0.842
N1 vs. N3a	1.632 (0.210–12.690)	0.640	0.216 (0.037–1.265)	0.089	0.747 (0.218–2.566)	0.643
N1 vs. N3b	0.587 (0.188–1.836)	0.360	0.371 (0.066–2.089)	0.261	0.514 (0.215–1.231)	0.135
Overall *P*	0.004		0.519		<0.001	
8th edition						
N category	HR (95%CI)	*P-*value	HR (95%CI)	*P-*value	HR (95%CI)	*P-*value
N1 vs. N0	2.095 (1.270–3.457)	0.004	1.722 (0.701–4.231)	0.236	1.700 (0.746–3.877)	<0.001
N1 vs. N2	1.212 (0.592–1.212)	0.599	1.985 (0.629–6.264)	0.242	1.098 (0.612–1.970)	0.754
N1 vs. N3	1.037 (0.649–1.656)	0.880	1.408 (0.701–2.829)	0.337	1.166 (0.818–1.662)	0.395
Overall *P*	0.008		0.316		0.001	

*Abbreviations*: *CI* Confidence interval. *HR* Hazard ratio. *OS* Overall survival. *DFS* Disease-free survival. *LRRFS* Locoregional recurrence-free survival. *DMFS* Distant metastasis-free survival

## Discussion

Based on our study findings, our data show that the 8th edition has a superior prognostic value for patients with NPC than the 7th edition.

In the treatment of NPC, IMRT has become the optimal radiation technique because of its clear advantage in target dose uniformity and better protection of adjacent organs at risk compared with 2-dimensional radiotherapy (2D-RT) or 3-dimensional conformal radiotherapy (3D-CRT). It can be administered in 2 ways, sequential technology (SEQ-IMRT) [16, 17] or SIB-IMRT [18]. Compared with SEQ-IMRT, SIB-IMRT simply uses a single radiation plan in the entire course of treatment, allowing the simultaneous delivery of different dose levels to different target volumes that reduces the treatment duration and enhances biologically equivalent dose (BED) [12, 19].

The TNM staging system is crucial for predicting prognosis, guiding treatment decisions for different risk groups, assessing treatment efficacy, and evaluating clinical outcomes between different centers. Therefore, the TNM staging system should be updated based on the development of radiation technology. The 7th staging system was based on the information data form the 2D-RT era, and several trials have been conducted to determine its value considering the advent of IMRT [20–22]. Zong [20] et al. analyzed the data of 1241 NPC patients treated with IMRT and revealed that the differences in LRRFS between T1 and T2, and between T2 and T3 were not significantly different (*P* = 0.055 and 0.605, respectively). Additionally, they reported that the hazard ratios for OS and disease-specific survival between T1 and T2 were not statistically significant. The study considered that the TNM staging system should downgrade stage T2 patients to T1 patients. In a study performed by Chen et al. [21] on 181 NPC patients with N0 stage, the authors reported that the difference in OS, LRRFS, and PFS between T1 and T2, and between T3 and T4 was not statistically significant. In this study, we also confirmed that there were no differences in OS, DFS, and DMFS between T1 and T2 (*P* = 0.987, 0.984, and 0.191). Fortunately, the 8th staging system was revised after the introduction of IMRT as a treatment option and several previous studies [11, 23–25] have reported its superiority over the 7th edition staging system. Our data confirmed that the 8th edition had better prognostic performance than the 7th edition.

For T categories, our data found that the T-classification in the 8th edition showed better separation between T3 and T2, and T3 and T1 compared to OS and LRRFS, while there were no significant differences in the T-classification in the 7th edition. A retrospective study performed by Pan et al. [11] on 1609 patients staged based on MRI findings and treated with IMRT at 2 major centers in Hong Kong and Mainland China (median follow-up of 5 years) found that there were statistically significant differences among OS between T3 and T2 (*P* = 0.009). Additionally, OuYang et al. [24] retrospectively studied 899 patients with NPC (from Hong Kong, Guangzhou, and Guangxi) who were staged based on MRI findings and received IMRT; this study compared the 7th and 8th staging systems and reported that the 8th edition had better differentiation of OS between T3 and T2 (*P* = 0.003). All these data confirmed that it was reasonable to downstage MP and LP from T4 in the 7th edition to T2 in the 8th edition. This change has increased the survival difference values between T3 and T2, and also resulted in improved classification of patients with NPC.

In terms of N categories, replacing SCF with the lower neck region to differentiate N1–2 and N3 is the main revision in the 8th edition. Ng et al. [10] first explored the possibility of replacing the SCF by levels IV and Vb as a demarcating criterion for the N3 category, and found this method potentially useful. A few studies debated that the definition of SCF involvement is primarily based on clinical examination and defining SCF using clinical landmarks is difficult [6–8]. However, the lower neck, as an anatomical landmark, can be reliably defined on the basis of both physical examination and cross-sectional images, thereby making it more convenient in clinical practice.

Several studies [23–25] have reported that the new staging system is useful in predicting outcomes with regard to N categories. In a study performed by Tang [23] that included 1790 NPC patients, the survival curves between different groups were accurately differentiated considering the 8th staging system. Another respective study also confirmed that the T-classification according to the 8th staging system showed better differentiation compared with that performed using the 7th edition [25]. Similarly, our results showed a clear difference between N2 and N3 among OS and DFS according to the new staging system. Moreover, we found no differences between N3a and N3b among OS, DFS, and DMFS considering the 7th staging system, indicating that this subgroup was unnecessary.

In terms of clinical stage, the 8th edition has upgraded IVC to IVB, and merged IVA and IVB from the 7th edition into IVA. Our data showed that the segregation of IVA and IVB in terms of survival was inaccurate in the 7th staging system as IVA and IVB share similar 5-year OS and DFS rates.

Our study included patients with NPC from a center between year 2009 and 2013 with a relative long follow-up time. However, because of the radiation technique, only 300 patients in our study underwent SIB-IMRT. This small number of patients may result in low end-point events that may weaken the power to convince the differences between both the staging systems. Another limitation was the nature of the study (retrospective).

## Conclusion

The 8th edition of the UICC/AJCC staging system has a higher prognostic value and better classification compared with the 7th edition considering SIB-IMRT as the latest treatment option.

## Supplementary Information

**Additional file 1.**

## Data Availability

The datasets analyzed during the current study are available from the corresponding author on reasonable request.
