# Supplementary material for: Comparing the 7th and 8th editions of UICC/AJCC staging system for nasopharyngeal carcinoma in the IMRT era
Source: BMC Cancer. 2021 Mar 30;21:327. doi: 10.1186/s12885-021-08036-8 (PMC8011200; doi:10.1186/s12885-021-08036-8)
Supplement: Supplementary file 1 — Additional file 1. [file 12885_2021_8036_MOESM1_ESM.docx]

**Comparing the 7th and 8th editions of UICC/AJCC staging system for nasopharyngeal carcinoma in the IMRT era**

Tao He^1,2,#^, Ruo-Nan Yan^1,2,#^, Hua-Ying Chen^1, #^, Yuan-Yuan Zeng^1,2,^ , Zhong-Zheng Xiang^1,2^, Fang Liu^1,2^, Bian-Fei Shao^1,2^, Jia-Chun Ma^1,2^, Xi-Ran Wang^1,2^, Lei Liu^1,2,*^

^1^ Department of Head and Neck Oncology, Cancer Center, State Key Laboratory of Biotherapy, West China Hospital, Sichuan University, Chengdu, Sichuan, PR China

^2^ Department of Radiation Oncology, Cancer Center, West China Hospital, Sichuan University, Chengdu, Sichuan, PR China

**^*^ Corresponding to:** Lei Liu.

Department of Head and Neck Oncology, Cancer Center, State Key Laboratory of Biotherapy, and the Department of Radiation Oncology, West China Hospital, Sichuan University, No. 37 Guo Xue Alley, Chengdu, Sichuan, PR China.

**Email:** liuleihx@gmail.com.

^#^ Tao He, Ruo-Nan Yan and Hua-Ying Chen contributed equally to this work.

supplement. Criteria of the 7th and 8th editions of the UICC/AJCC staging system for nasopharyngeal carcinoma.

|  | 7th edition | 8th edition |
| --- | --- | --- |
| T category |  |  |
|  | T1: Nasopharynx, oropharynx, or nasal cavity without parapharyngeal extension | T1: Nasopharynx, oropharynx, or nasal cavity without parapharyngeal extension |
|  | T2: Parapharyngeal extension | T2: Parapharyngeal extension, adjacent soft tissue involvement (medial pterygoid, lateral pterygoid, prevertebral muscles) |
|  | T3: Bony structures of skull base and/or paranasal sinuses | T3: Bony structures (skull base, cervical vertebra) and/or paranasal sinuses |
|  | T4: Intracranial, cranial nerves, hypopharynx, orbit, infratemporal fossa/masticator space | T4: Intracranial extension, cranial nerve, hypopharynx, orbit, extensive soft tissue involvement (beyond the lateral surface of the lateral pterygoid muscle, parotid gland) |
| N category |  |  |
|  | N0: No regional lymph node metastasis | N0: No regional lymph node metastasis |
|  | N1: Unilateral cervical, unilateral or bilateral retropharyngeal lymph nodes above the supraclavicular | N1: Retropharyngeal (regardless of laterality)  Cervical: unilateral, ≤6cm, and above caudal border of cricoid cartilage |
|  | N2: Bilateral metastasis in lymph nodes, ≤6cm in greatest dimension, above the supraclavicular fossa | N2: Cervical: bilateral, ≤6cm, and above caudal border of cricoid cartilage |
|  | N3a: ＞6cm in dimension | N3: ＞6cm and/or below caudal border of cricoid cartilage (regardless of laterality) |
|  | N3b: Supraclavicular fossa |  |
| Clinical stage |  |  |
|  | I: T1N0M0 | I: T1N0M0 |
|  | II: T2N0-1M0, T1NIM0 | II: T2N0-1M0, T1NIM0 |
|  | III: T1-2N2M0, T3N0-2M0 | III: T3N0-2M0, T1-2N2M0 |
|  | IVA: T4N0-2M0 | IVA: T4 or N3M0 |
|  | IVB: Any T1-4N3M0 | IVB: Any T, any N M1 |

Abbreviations: UICC/AJCC, Union for International Cancer Control/American Joint Committee on Cancer.
